# Supplementary material for: Meiotic Nuclear Architecture in Distinct Mole Vole Hybrids with Robertsonian Translocations: Chromosome Chains, Stretched Centromeres, and Distorted Recombination
Source: Int J Mol Sci. 2020 Oct 15;21(20):7630. doi: 10.3390/ijms21207630 (PMC7589776; doi:10.3390/ijms21207630)
Supplement: Supplementary file 1 [file ijms-21-07630-s001.pdf]

Supplementary materials

Meiotic nuclear architecture in distinct mole vole hybrids with Robertsonian translocations: chromosome chains, stretched centromeres, and distorted recombination

Sergey Matveevsky [1,*], Artemii Tretiakov [1], Anna Kashintsova [1], Irina Bakloushinskaya [2], Oxana Kolomiets [1]

[1] Vavilov Institute of General Genetics, Russian Academy of Sciences, 119991 Moscow, Russia; temanch@gmail.com (A.T.), koly4kina2009@mail.ru (A.K.), olkolomiets@mail.ru (O.K.)

[2] Koltzov Institute of Developmental Biology, Russian Academy of Sciences, 119334 Moscow, Russia; irina.bakl@gmail.com (I.B.)

* Correspondence: sergey8585@mail.ru, s.matveevsky@vigg.ru

Table S1. Total meiotic data of Ellobius species and hybrids

		1	2	3	4	5	6	7	8
	**Mole voles**	**Total number of pachytene nuclei**	**Number of cells with moved XX to the periphery of the nucleus, M±SD, % (*n* nuclei) [Fig 2I]**	**Open XX bivalents, M±SD, % (*n* nuclei) [Fig 2J]**	**Number of nuclei with associations of XX and SC trivalents (in hybrids) or XX and autosomes, M±SD, % (*n* nuclei) [Fig 2K]**	**Number of nuclei without free closed SC trivalents (SC trivalents in the chains), M±SD, % (*n* nuclei) [Fig 3E]**	**Number of nuclei with stretched centromeres, M±SD, % (*n* nuclei) [Fig 3I]**	**Number of nuclei with chromosome #7 detected, M±SD, % (*n* nuclei) [Fig 3L]**	**Average number of MLH1 foci per nucleus, M±SD (*n* nuclei) [Fig 5F]**
**A**	***E. talpinus***, ♂, 2n=54, NF=56 (N=3)	253	**0.73±0.03,** 72.59% (*n*=142)	**0.09±0.03**, 9.85% (*n*=140)	0	–	0	always	**23.48±3.3** (*n*=101)
**B**	***E. tancrei***, ♂, 2n=54, NF=56 (N=2)	153	**0.72±0.07,** 72.44% (*n*=132)	**0.11±0.07**, 10.92% (*n*=132)	**0.06±0.04***, 5.9% (*n*=132)	–	0	always	**23.1±3.1** (*n*=99)
**C**	***E. tancrei***, ♂, 2n=34, NF=56 (N=5)	241	**0.76±0.09,** 76.48% (*n*=98)	**0.09±0.02**, 8.84%, (n=98)	**0.02±0.03**, 2.25%, (*n*=98)	–	0	always	**22.8±3.7** (*n*=151)
**D**	Hybrid F1 ***E. tancrei X E. tancrei***, ♂, *2n=44, NF=56* (N=3)	168	**0.50±0.11,** 49.9% (*n*=108)	**0.53±0.11**, 52.97% (*n*=114)	**0.50±0.08**, 50.37% (*n*=115)	**0.09±0.02**, 9.42% (*n*=99)	**0.16±0.06**, 15.72% (*n*=96)	**0.94±0.02**, 93.59% (*n*=78)	**15.4±4.4** (*n*=47)
**E**	Hybrid F1 ***E. talpinus X E. tancrei***, ♂, *2n=44, NF=55 (N=5)*	198	**0.49±0.13,** 48.61% (*n*=172)	**0.55±0.15**, 55.6% (*n*=113)	**0.45±0.18**, 45.34% (*n*=98)	**0.78±0.12**, 77.95% (*n*=102)	**0.69±0.11**, 69.52% (*n*=105)	**0.91±0.09**, 91.17% (*n*=102)	**14.8±4.7** (*n*=94)
		**Significant ($P < 0.05$)**	A/D, A/E, B/D, B/E, C/D, C/E	A/D, A/E, B/D, B/E, C/D, C/E	B/D, B/E, C/D, C/E	D/E	D/E		A/E, B/E, C/E, A/D, B/D, C/D, D/E
		**Not significant**	A/B, A/C, B/C, D/E	A/B, A/C, B/C, D/E	B/C, D/E			D/E	A/C, A/B, B/C
		**Comments**			* We did not include data from the Matveevsky et al., 2015, in which this indicator is 0.17 ± 0.03. We used a different way of counting.				

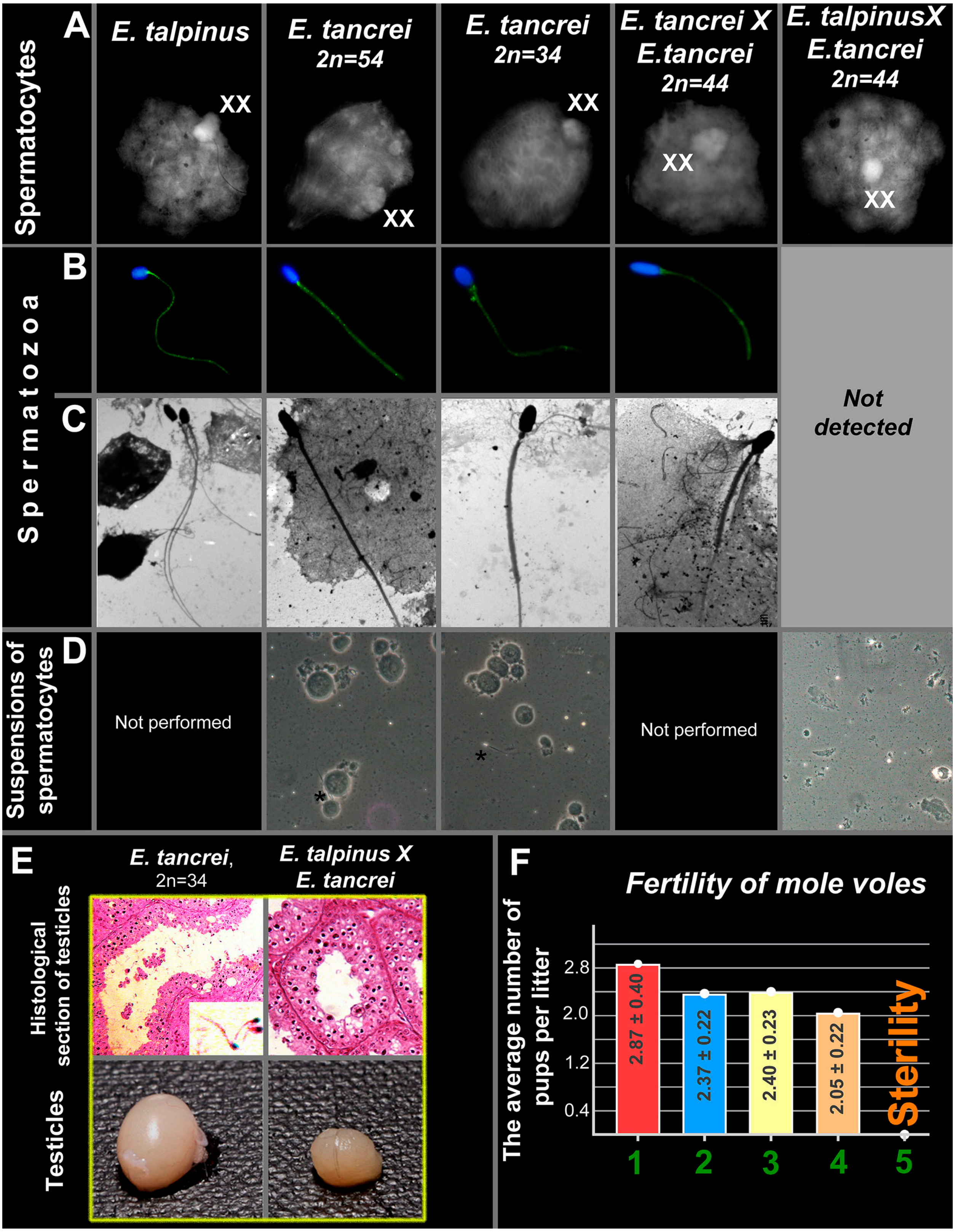

Figure S1. Fertility parameters of *Ellobius* species and hybrids. (A) DAPI-stained spermatocytes. **(B, C)** Spermatozoa: light (B) and electron (C) micrographs. Spermatozoa in B were detected by nonspecific immunostaining (SYCP3 antibody, green) and DAPI-staining (blue). **(D)** Suspension of spermatocytes. **(E)** Comparison of histological sections of testes and the size of testes in *Ellobius tancrei* (on the left) and the interspecific hybrid (on the right). **(F)** The average number of pups per litter in parents and hybrids: 1. *Ellobius talpinus*; 2. *E. tancrei* (2n = 54); 3. *E. tancrei* (2n = 34); 4. *E. tancrei* × *E. tancrei* hybrid; 5. *E. talpinus* × *E. tancrei* hybrid.

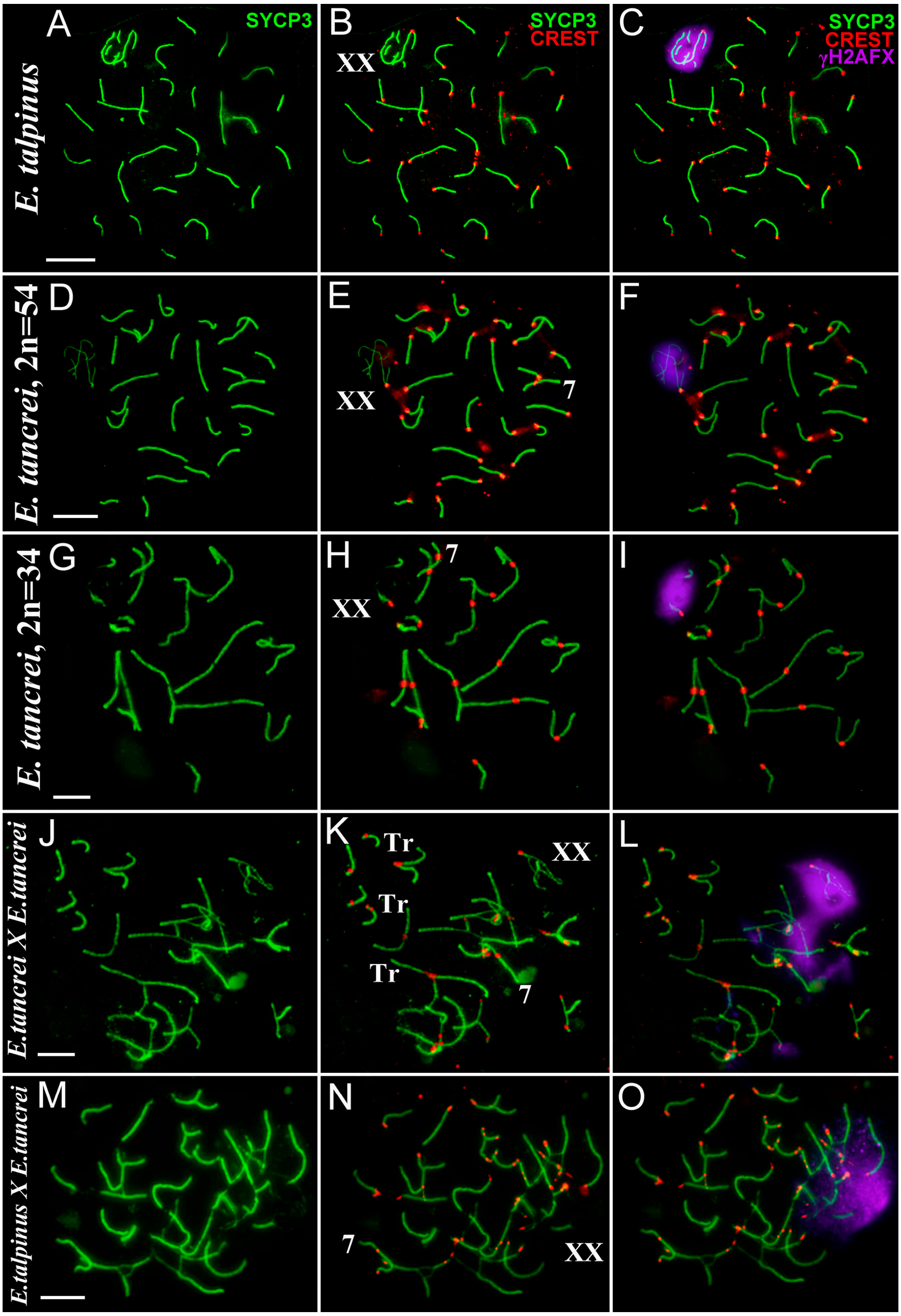

Figure S2. Pachytene spermatocytes of *Ellobius* species and hybrids. Synaptonemal complexes (SC)s were immunostained with antibodies against SYCP3 (green) and centromeres—with an antibody to kinetochores (CREST, red). An anti-γH2AFX (violet) antibody was used as a marker of chromatin inactivation. XXs were moved to the periphery of the nuclei and were covered by γH2AFX-cloud in parent species **(A–C**, **D–F**, and **G–I)**. The γH2AFX-cloud covered the XX bivalent and spread to some asynaptic segments in both 44-chromosome hybrids **(J–L** and **M–O)**. Scale bars represent 5 μm.

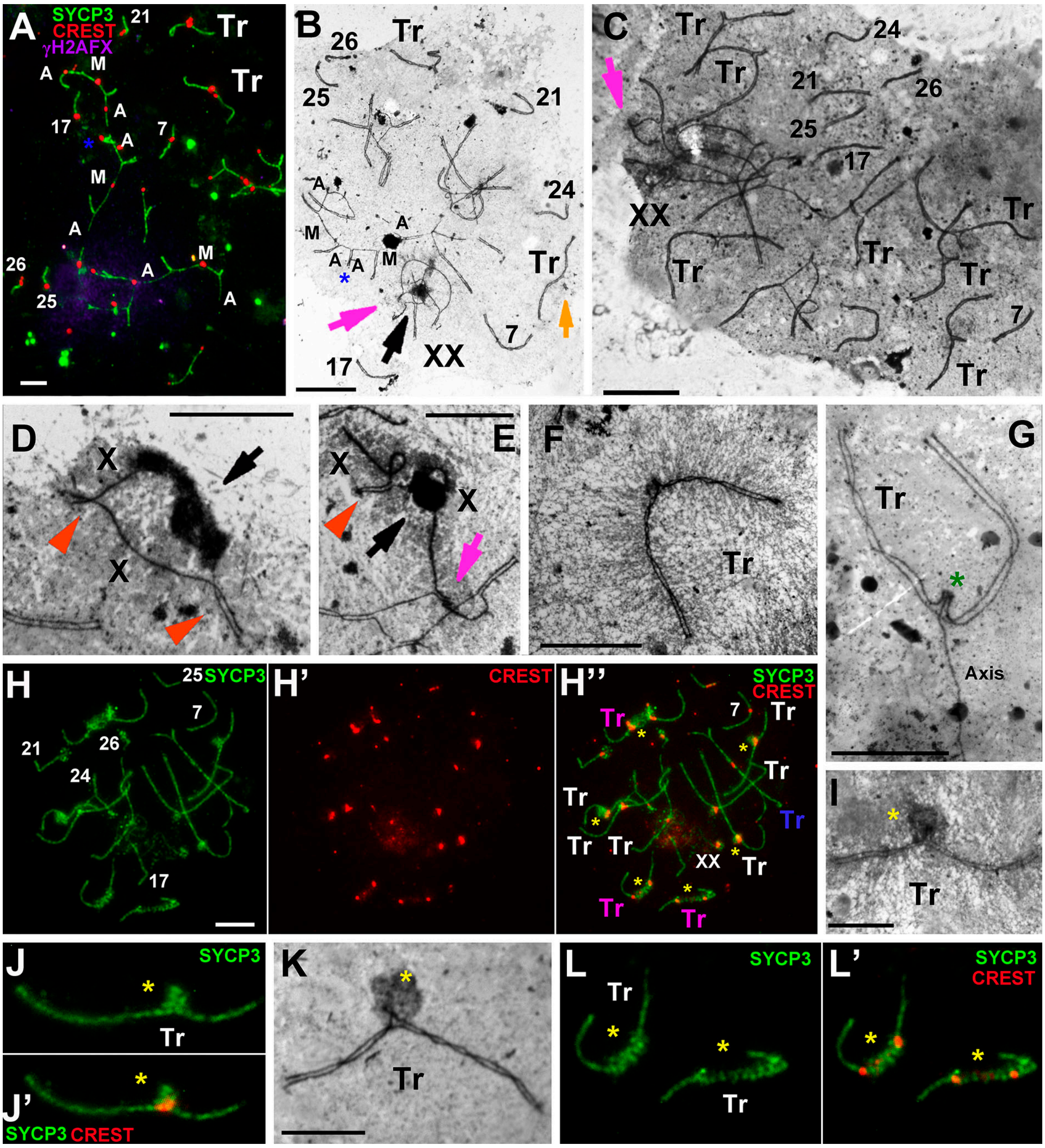

Figure S3. Synaptic behavior of chromosomes in intraspecific *E. tancrei* × *E. tancrei* hybrid. Light micrographs after immunostaining (A, H, J, and L). SCs were immunostained with antibodies against SYCP3 (green) and centromeres—with an antibody to kinetochores (CREST, red). An anti-γH2AFX (violet) antibody was used as a marker of chromatin inactivation. Electron micrographs after $AgNO_3$-staining (B-G, I, and K). The numbers of autosomes (B, C, and H) correspond to the chromosome number of the karyotype (see Fig 1E). Orange arrowheads show synaptic sites of the sex (XX) bivalent. Black arrows show chromatin dense body (ChB) of the XX (B, D, and E). Tr indicates trivalent, M indicates metacentric, and A indicates acrocentric. **(A, B)** SC trivalents (A/M/A) chains joined together by short arms of nonhomologous acrocentrics at the early-to-mid pachytene stage (blue stars). XX bivalents have been associated with SC trivalents (Trs) or bivalents (see the violet signal in A and pink arrow in B). Both nuclei had two free Trs. The light orange arrow shows atypical prolonging of short arms of acrocentrics of the SC trivalent (B). **(C)** Seven free and closed SC Trs were formed at the late pachytene stage. XX bivalents were associated with SC chains (pink arrow). **(D)** Closed sex bivalent with two synaptic sites at the ends and a central unpaired region at the periphery of the nucleus. A long ChB was observed along one of the axes (black arrow). **(E)** One of the X chromosomes axes was in contact with the SC trivalent (pink arrow). **(F)** A closed SC trivalent with well-structured chromatin. **(G)** Triple synapsis in the region of short arms of a SC trivalent (green star). **(H, J, K,** and **L)** The only case of the nucleus with all 10 trivalents (H). SYCP3- and $AgNO_3$-positive dense material was revealed in the region of short arms of some SC trivalents (yellow stars; white "Tr" in H; I–K). Some trivalents had a stretched metacentric, in the stretch segment of which SYCP3- positive dense material was also observed. Most nuclei had no such features in hybrid spermatocytes (L, L'). Scale bars represent 5 μm.

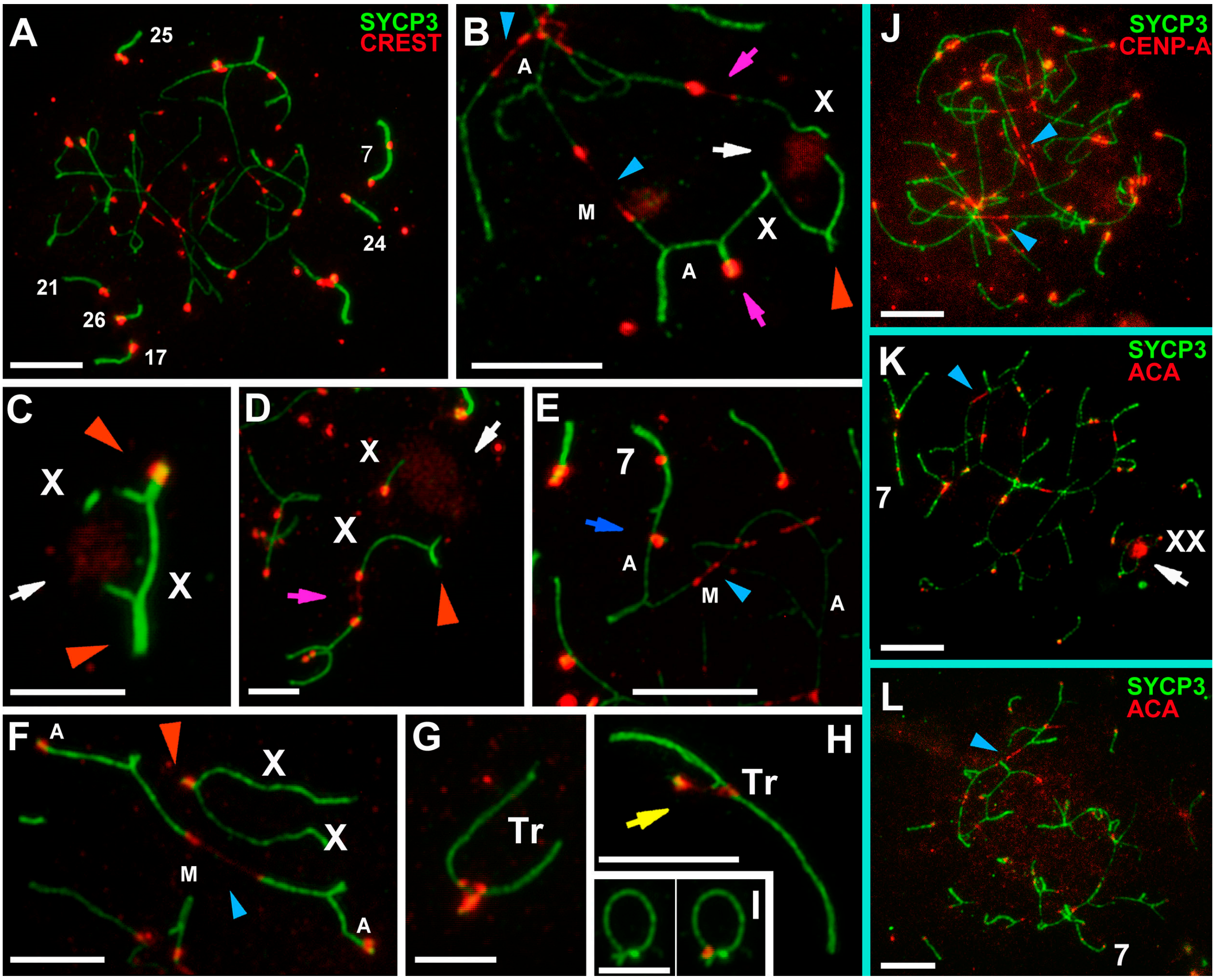

Figure S4. Synaptic behavior of chromosomes in interspecific *E. talpinus* × *E. tancrei* hybrid. SCs were immunostained with antibodies against SYCP3 (green) and centromeres—with antibodies to kinetochores (ACA, CREST, or CENP-A, red). The numbers of autosomes (A) correspond to the chromosome number of the karyotype (Fig 1D). Orange arrowheads show synaptic sites of the sex (XX) bivalent (B, C, D, and F). White arrows show chromatin dense body (ChB) of the XX (b, c, d, and k). Tr – indicates trivalent, M indicates metacentric, and A indicates acrocentric. **(A)** There were no free SC trivalents in the pachytene nucleus; all SC trivalents were in chains. The XX bivalent is not visible. **(B)** Double association of the XX bivalent with SC trivalents. An X univalent was associated with a SC trivalent by a thin CREST-positive line. **(C)** The XX bivalent with two synaptic sites and ChB. **(D)** An open XX bivalent with ChB. One of X univalent was associated with SC trivalent by a thin CREST-positive line. **(E)** The only case of the association of chromosome #7 with a SC trivalent. An acrocentric homolog of chromosome #7 involved in an association (blue arrow). **(F)** An open XX bivalent and SC trivalent with a stretched centromere. **(G)** A free closed SC trivalent. **(H)** The yellow arrow shows atypical lengthening of short arms of acrocentrics of the SC trivalent (H). **(I)** A ring-like univalent. **(J–L)** CENP-A-positive (J) and ACA-positive (K and L) centromeric region of metacentrics in some SC trivalents were stretched. There were no free SC trivalents. Scale bars represent 5 μm.

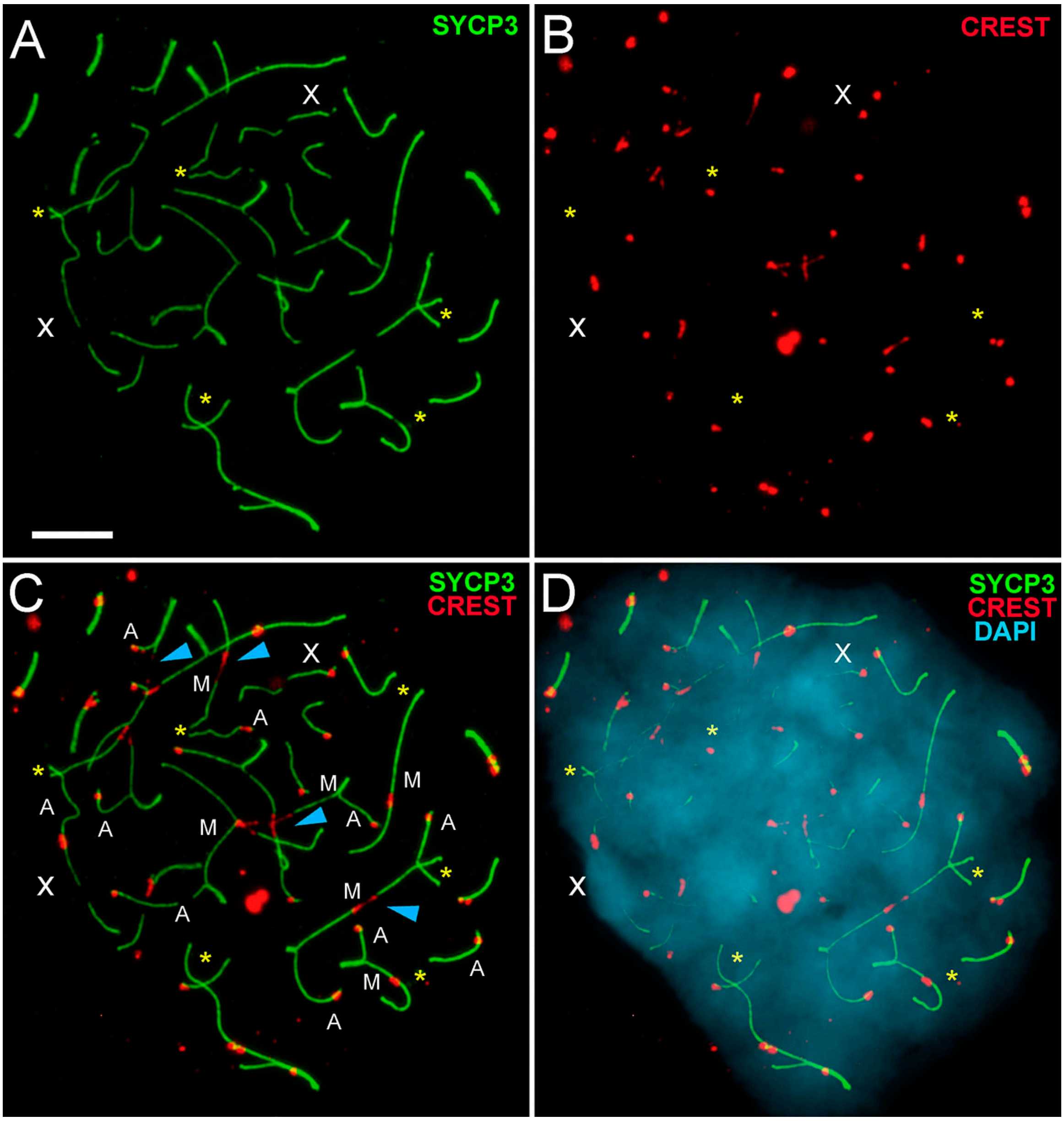

Figure S5. Synaptic behavior of chromosomes in interspecific *E. talpinus* × *E. tancrei* hybrid at zygotene nucleus. (A–D) SCs were immunostained with antibodies against SYCP3 (green) and centromeres—with an antibody to kinetochores (CREST, red). DAPI stained chromatin (blue). M indicates metacentric and A indicates acrocentric. Acrocentrics and metacentrics in SC trivalents adjusted together. Some acrocentrics and some arms of metacentrics had already entered synapsis, and some were just starting the synapsis process (yellow stars). Some metacentrics of SC trivalents had stretched centromeres (blue arrowheads). Sex chromosomes did not enter synapse and were located as X univalents. Their identification was possible based on length and a similar scenario of the behavior of axial elements, DAPI-positive signals, chromatin-dense body (ChB), and comparison with other micrographs (see Fig 2H). Scale bars represent 5 μm.

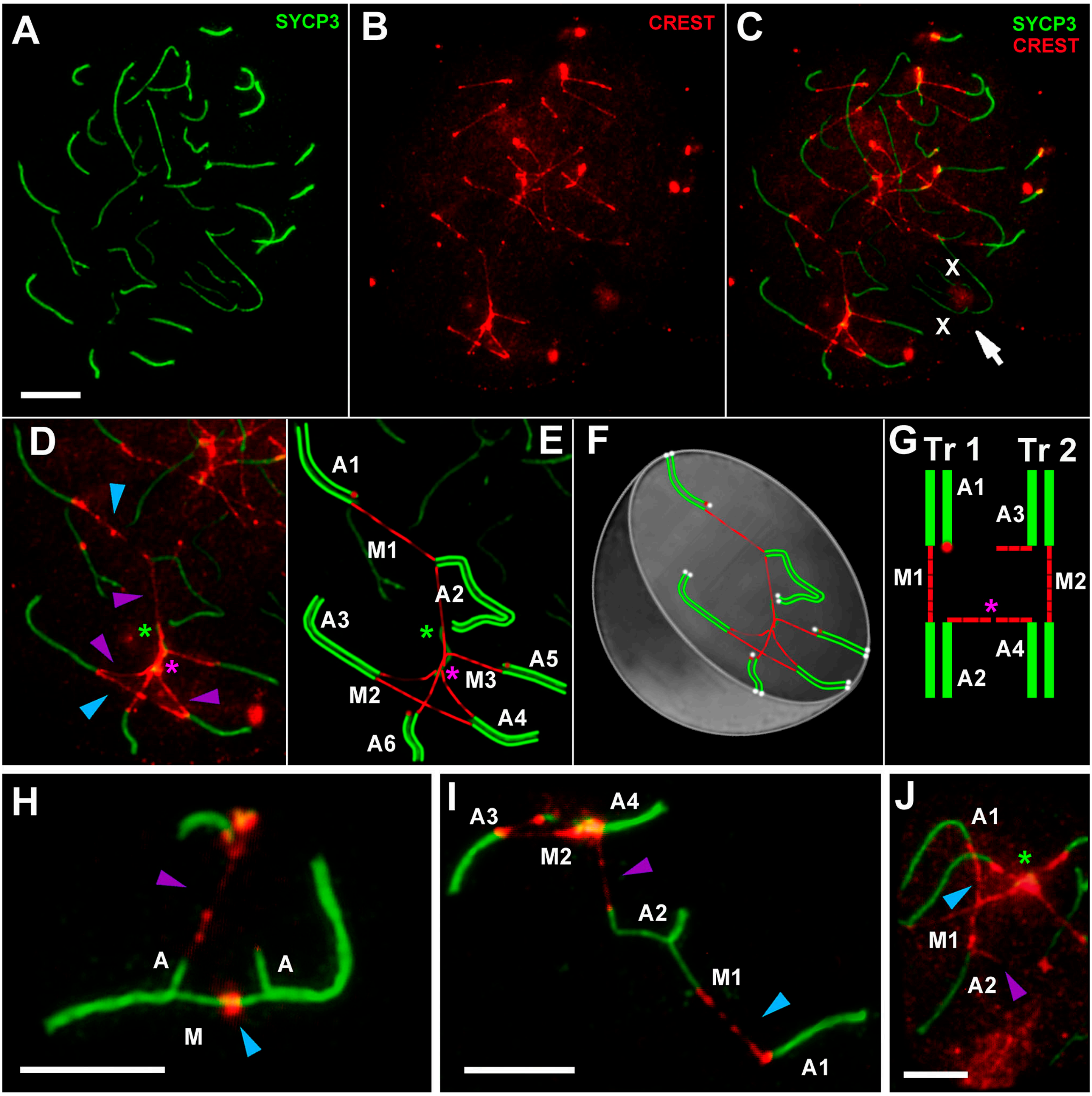

Figure S6. SC trivalents and stretched centromeres in interspecific *E. talpinus* × *E. tancrei* hybrid. SCs were immunostained with antibodies against SYCP3 (green) and centromeres—with an antibody to kinetochores (CREST, red). Blue arrowheads show centromeric regions of metacentrics in SC trivalents. Violet arrowheads show centromeric regions of acrocentric in SC trivalents. The white arrow shows a chromatin dense body (ChB) of the XX bivalent. Green stars show interlocking points. Tr indicates trivalent, M indicates metacentric, and A indicates acrocentric. **(A–C)** Pachytene nucleus: SYCP3-fragments (pseudobivalents) of SC trivalents were distant from each other (A). These fragments were connected by extensive stretched centromeric regions (B, C). **(D-G)** Part of the nucleus in A–C: Tree SC trivalents were linked by stretched centromeres of metacentrics and acrocentrics. SYCP3-segment of axial elements (green star) was an interlocking point. The scheme of the SC trivalent chains (E) and simulation of this chain in the nucleus (F) are presented. White dots at the chromosome ends mark the attachment points of the chromosomes to the nuclear envelope (F). Two SC trivalents A1/M1/A2 and A3/M2/A4 are connected by stretched centromeres of acrocentrics (pink stars) (D–G). **(H–J)** Examples of SC trivalents with stretched centromeres of metacentrics and acrocentrics. Scale bars represent 5 µm

.

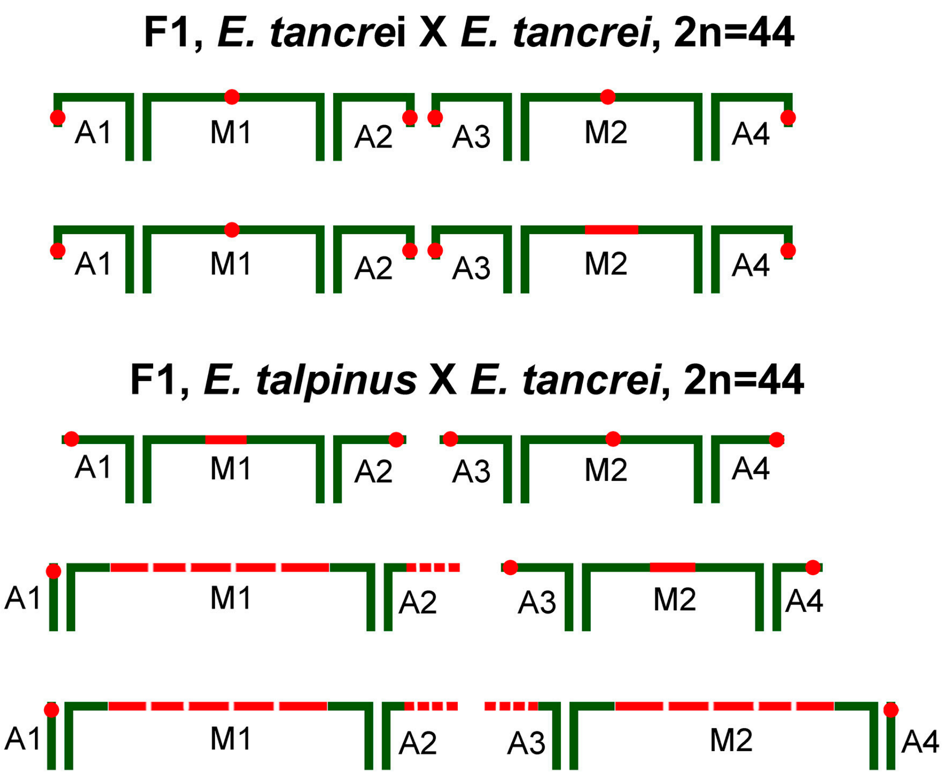

Figure S7. Types of SC trivalent chains in *Ellobius* hybrids. Red dashed inserts correspond to centromere stretching. Green lines correspond to the axial elements. See explanations in the text.

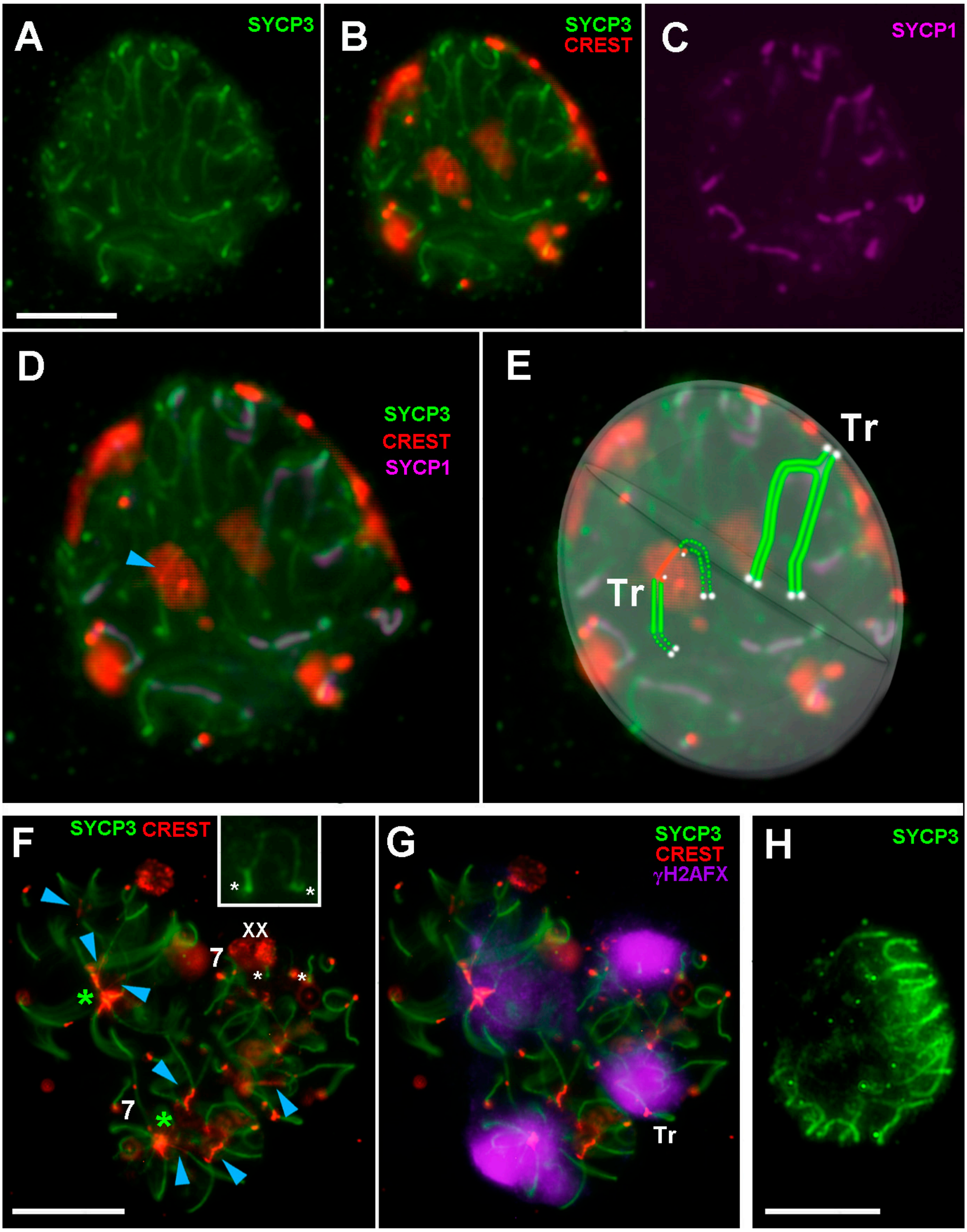

Figure S8. Spermatocyte squashes of interspecific *E. talpinus* × *E. tancrei* hybrid. Axial elements were identified using anti-SYCP3 antibodies (green), a central element using anti-SYCP1 (magenta), and an anti-CREST antibody for kinetochores (red). An anti-γH2AFX (violet) antibody was used as a marker of chromatin inactivation. DAPI stained chromatin (blue). Blue arrowheads show stretched centromeric regions in SC trivalents. One of the SC trivalent had a stretched centromere. **(A–E)** The short arms of the acrocentrics in the SC trivalents were attached to the nuclear envelope. The central element (SYCP1) was formed in the SC trivalent. White dots at the chromosome ends mark the attachment points of the chromosomes to the nuclear envelope (E). **(F, G)** Three squashes with some stretched centromeres of a SC trivalent. One of the squashes had a closed XX bivalent covered by a γH2AFX cloud. White stars show synaptic sites of the XX bivalent (enlarged in inset). Some open SC trivalents were also covered by a γH2AFX cloud (G). Some SC trivalents had a centromeric link in interlocking point (green stars) (F). **(H)** Zygotene-like stage: Chromosome–nuclear envelope interactions were visible. Scale bars represent 5 μm.

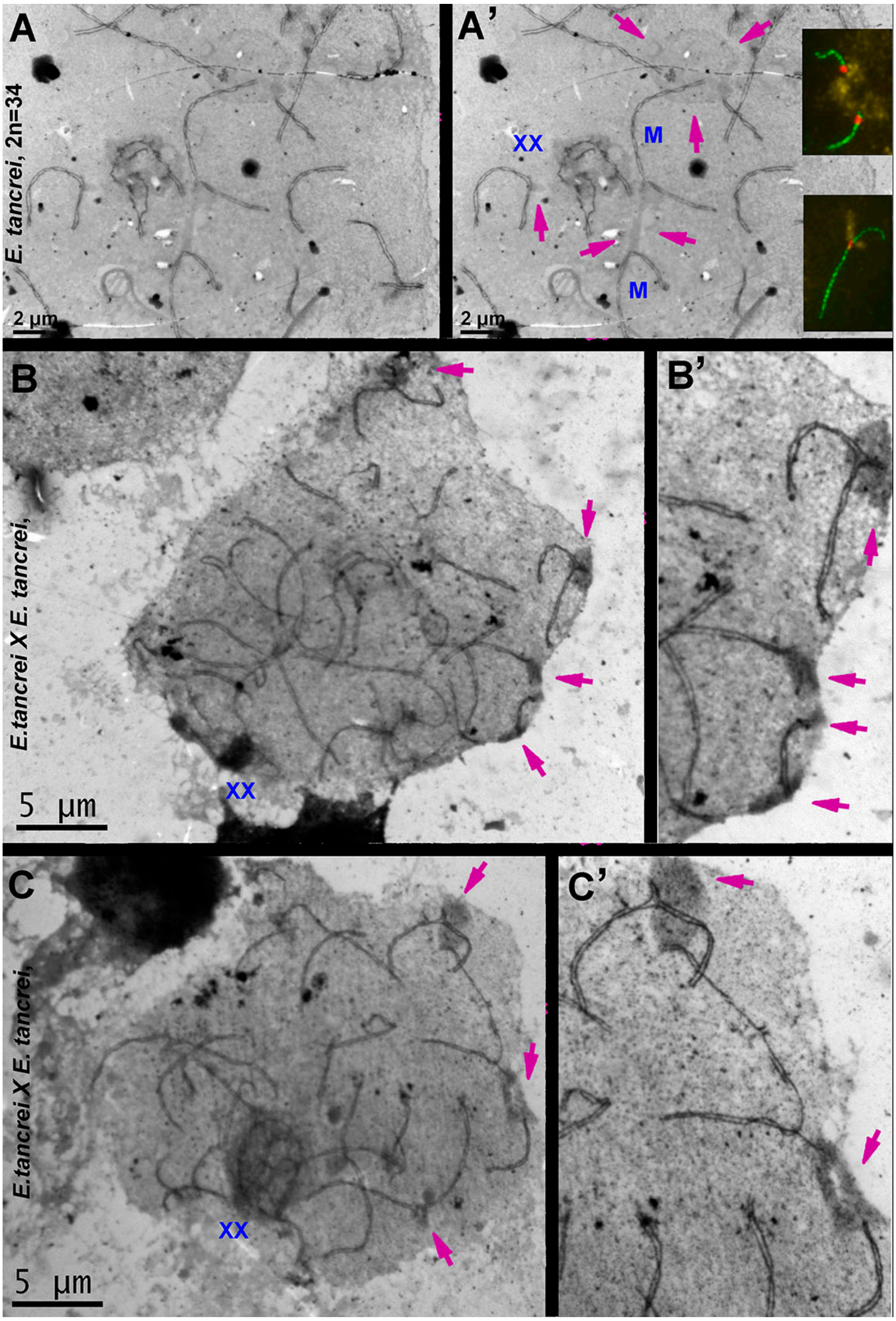

Figure S9. Heterochromatic links of chromosomes and nuclear envelope. Pink arrows show heterochromatin mass of chromosomes and SC trivalents. **(A, A')** Part of the nucleus of *E. tancrei* (2n = 34) at the early pachytene stage. The linear heterochromatin link between two metacentrics (M) and cloud-like heterochromatic mass is visible. (Insets) SCs were immunostained by antibodies against SYCP3 (green), heterochromatin—by an H3K9me3 antibody (yellow)—and centromeres—by an antibody to kinetochores (CREST, red). H3K9me3 foci are cloud-like signals near the centromeres of acrocentrics (top inset) and linear signal near the centromere of the submetacentric (bottom inset). **(B, C)** Pachytene nuclei of the *E. tancrei* × *E. tancrei* hybrid. Some closed SC trivalents have a heterochromatic spot in the short arms of acrocentrics linked with the nuclear envelope (B', C'). Heterochromatic spots of some acrocentrics have links with the nuclear envelope too (B', C').
